# Genomic imbalances of chromosome 15 in patients with autistic features and global developmental delay

**DOI:** 10.1192/j.eurpsy.2022.582

**Published:** 2022-09-01

**Authors:** M. Budisteanu, S. Papuc, A. Erbescu, L. Albulescu, A. Arghir

**Affiliations:** 1 Prof. Dr. Alex. Obregia Clinical Hospital of Psychiatry, Psychiatry Research Laboratory, Bucharest, Romania; 2 Victor Babes National Institute of Pathology, Medical Genetics, Bucharest, Romania

**Keywords:** developmental delay, chromosome 15, genomic imbalances, autism

## Abstract

**Introduction:**

Background: Copy-number variants (CNVs) of chromosome 15 have been associated with neurodevelopmental disorders like autism spectrum disorders (ASDs) and developmental delay.

**Objectives:**

We report 6 patients with autistic features and other neurodevelopmental problems carrying CNVs of chromosome 15.

**Methods:**

Materials and methods: The probands belong to a group of patients referred to our clinic and laboratory with autism as main feature. A complete clinical evaluation was performed with focus on neurologic, psychiatric, and psychological evaluation with specific autism tests. Array-based comparative genomic hybridization (array-CGH) was performed using 180K platform (Agilent technology).

**Results:**

six patients investigated by array-CGH had a CNV involving chromosome 15. Four of these patients, previously reported by us (ref 1), had small duplications of 15q13.3 involving *CHRNA7* and *OTUD7A* genes. The other two patients had large deletions of 15q21q22 and 15q24, respectively. A deletion of 15q21.2 - q22.2 was detected in one patient. The deleted region contains 62 genes and has been rarely reported in patients with neurodevelopmental disorders. A deletion of 15q24.1 - q24.2 was detected in the other patient. This region is recurrently deleted in developmentally delayed patients (ref 3).

**Conclusions:**

Our data highlight that chromosome 15 is a hub for neurodevelopmental disorder and illustrates the utility of array-CGH in the investigation of patients with autism, specifically in the context of complex phenotypes. Acknowledgment: The research leading to these results has received funding from the EEA Grant 2014-2021, under the project contract No 6/2019. References: Genes (Basel). 2021 Jul 1;12(7):1025. https://www.omim.org/entry/618060 Clinical Genome Resource. https://dosage.clinicalgenome.org/clingen_region.cgi?id=ISCA-46296

**Disclosure:**

No significant relationships.

